# Hepatic *Chemerin* and *Chemokine-Like Receptor 1* Expression in Patients with Chronic Hepatitis C

**DOI:** 10.1155/2014/517820

**Published:** 2014-07-10

**Authors:** Michał Kukla, Brygida Adamek, Marek Waluga, Marzena Zalewska-Ziob, Janusz Kasperczyk, Andrzej Gabriel, Włodzimierz Mazur, Barbara Sobala-Szczygieł, Rafał J. Bułdak, Wojciech Zajęcki, Lucjan Kępa, Katarzyna Ziora, Krystyna Żwirska-Korczala, Andrzej Wiczkowski, Marek Hartleb

**Affiliations:** ^1^Department of Gastroenterology and Hepatology, Medical University of Silesia, Ulica Medyków 14, 40-752 Katowice, Poland; ^2^Department of General Biology, Medical University of Silesia, Katowice, Poland; ^3^Department of Environmental Medicine and Epidemiology, Medical University of Silesia, Katowice, Poland; ^4^Department of Pathomorphology in Zabrze, Medical University of Silesia, Katowice, Poland; ^5^Department of Infectious Diseases in Chorzów, Medical University of Silesia, Katowice, Poland; ^6^Department of Infectious Diseases in Bytom, Medical University of Silesia, Katowice, Poland; ^7^Department of Physiology in Zabrze, Medical University of Silesia, Katowice, Poland; ^8^Department of Paediatrics in Zabrze, Medical University of Silesia, Katowice, Poland

## Abstract

*Introduction*. Chemerin seems to be involved in pathogenesis of chronic hepatitis C (CHC). Hepatic expressions of chemerin and its receptor, chemokine receptor-like 1 (CMKLR1), in CHC have not been studied so far. * Aim*. To evaluate * chemerin* and * CMKLR1* hepatic expression together with serum * chemerin* concentration in CHC patients and to assess their relationship with metabolic and histopathological abnormalities. * Methods*. The study included 63 nonobese CHC patients. Transcription of * chemerin* and * CMKLR1* was assessed by quantitative real-time PCR, while serum chemerin was assessed by enzyme-linked immunosorbent assay. * Results*. Expression of * chemerin* and * CMKLR1* was present in the liver of all CHC patients regardless of sex or age. This expression was not associated with necroinflammatory activity and steatosis grade, fibrosis stage, and metabolic abnormalities. There was a negative association between serum chemerin and * chemerin* hepatic expression (*r* = (−0.41), *P* = 0.006). * Conclusion*. The study for the first time confirmed a marked expression of * chemerin* and * CMKLR1* in the liver of CHC patients. The study was performed using the homogenates of human liver tissue, so it is not possible to define whether hepatocytes or other cell types which are abundantly represented in the liver constitute the main source of * chemerin* and * CMKLR1* mRNA.

## 1. **Introduction**

Chemerin, a member of the adipocytokines family, is also known as* tazarotene-induced gene 2* (*TIG2*) or retinoic acid receptor responder protein 2 (RARRES2) [1, 2]. It was first discovered as a chemotactic peptide directing macrophages and dendritic cells toward sites of inflammation, being involved in both adaptive and innate immunity [3–5]. Interest in chemerin has grown since it was discovered in fat tissue as a novel adipokine secreted by adipose tissue [6]. Chemerin has been associated with autocrine/paracrine signaling for adipocyte differentiation and maturation as well as with glucose uptake and lipolysis stimulation in adipocytes [7–9]. Human chemerin is a protein secreted in an inactive form as prochemerin and activated by inflammatory and coagulation serine proteases [10]. Hence, local and systemic levels of bioactive chemerin depend on proteolytic processing and are not simply related to chemerin protein concentrations [11, 12]. Serum circulating form, identified by Meder and co., is composed of 134 amino acids, truncated in N- and C-terminus compared with precursor [13]. Its levels in humans are related to body mass index (BMI), concentration of triglycerides and total cholesterol, and levels of blood pressure and insulin resistance (IR) [7, 14, 15]. However, chemerin levels higher in hepatic vein blood samples than in systemic circulation indicate that it is synthesized and secreted by the liver [16]. In humans, chemerin mRNA was found to be highly expressed not only in white adipose tissue, but also in liver and lungs [7]. Chemerin exerts its functions by binding the G protein-coupled receptor, chemokine receptor-like 1 (CMKLR1) (also known as chemerin receptor 23 (ChemR23)) [2, 17]. Its expression has been defined in various leukocyte populations anda number of other cell types including preadipocytes and adipocytes, osteoclasts, chondrocytes, skeletal muscle cells, and endothelial cells [18–21].

Some of the clinical and pathological features of hepatitis C virus- (HCV-) induced chronic hepatitis (chronic hepatitis C, CHC) indicate that HCV may contribute to a wide spectrum of metabolic disturbances, including insulin resistance, increased prevalence of impaired glucose tolerance, and lipid metabolism abnormalities [22–24]. Steatosis has been recognized as one of the factors capable of influencing liver fibrosis, inflammation, and angiogenesis [25–27] and the “metabolic” approach in patients with CHC was postulated [28]. These observations triggered exploration of adipocytokines contribution in CHC pathophysiology [29–32]. We previously found serum chemerin to be significantly higher in CHC patients compared to controls and negatively associated with necroinflammatory grade [33]. Thus, we decided to analyze* chemerin* and its receptor,* CMKLR1*, liver tissue expression together with serum chemerin concentrations in CHC patients and search for their relationships with metabolic disorders and histopathological abnormalities.

## 2. Materials and Methods

### 2.1. Patient Selection and Serological Assays

The study was performed on 63 nonobese patients with CHC (29 men/34 women), with body mass index (BMI) ≥19 or ≤30 kg/m^2^, infected with the HCV genotype 1b, aged between 19 and 70 years—average 46.6 ± 14.6 years. The diagnosis of CHC was confirmed by the presence of serum HCV-RNA assayed with the reverse transcription polymerase chain reaction (RT-PCR) method (Amplicor Roche/Promega v.2 Diagnostic Test, Branchburg, NJ, USA). Virus genotype was assessed by a reverse-hybridization line probe assay (LiPA Versant Test, Milwaukee, WI, USA) and viral load by signal amplification nucleic acid probe assay for the quantitation of human hepatitis C viral RNA (Bayer Versant_HCV RNA 3.0 Assay (bDNA); Bayer Diagnostics, Berkeley, CA, USA). All patients were naive for the antiviral treatment. Exclusion criteria included other virus genotypes; drug or alcohol abuse; autoimmune, neoplastic, thyroid, and psychiatric diseases; hepatitis B or HIV coinfection; diabetes mellitus; renal or heart failure.

The control group consisted of 30 healthy volunteers (15 males and 15 females) aged 47.9 ± 14.8 years (males: 44.7 ± 14.9)/(females: 51.1 ± 14.5 years) without anti-HCV antibodies, hepatitis B surface (HBs) antigen, and HIV negative and with alcohol consumption less than 20 g/day and normal alanine aminotransferase (ALT) activity. This group of study participants had BMI 23.9 ± 3.3 kg/m^2^, range 19.3–27.0 kg/m^2^.

Patients of the study group had their systolic and diastolic blood pressure and waist circumference measured. For further analysis we defined two subgroups: BMI <25 and BMI ≥25 kg/m^2^. On the day of liver biopsy, a single blood sample was drawn in the morning from all patients subjected to fasting. In healthy volunteers blood samples were collected in fasting state. Control group did not undergo liver biopsy.

The samples were centrifuged, and serum was aliquoted and frozen at –70°C until further processing. All the study participants underwent oral glucose tolerance test for diagnosis of diabetes mellitus or impaired glucose tolerance.

The study protocol was approved by the Ethical Committee of the Medical University of Silesia in Katowice, Poland, and conformed to the ethical guidelines of the Declaration of Helsinki. Informed consent was obtained from all of the study participants.

Chemerin serum concentration was assessed in duplicate by immunoenzymatic method with the commercially available Human Chemerin ELISA Kit, Catalogue number E0945h; Wuhan Uscn Sciences Co. Ltd., China. The study evaluated full-length form of chemerin. Insulin concentration was measured by Diametra Insulin EIA Kit, Catalogue number DKO076; Diametra S.r.l headquarter: via Garibaldi, Foligno (PG), Italy. The remaining biochemical parameters were measured using routine methods. The upper limit of ALT activity was set at 38 IU/L and aspartate aminotransferase (AST) at 40 IU/L, while gamma-glutamyltransferase (GGTP) activity was set at 50 IU/L and bilirubin serum concentration at 17 *μ*mol/L. The degree of IR was calculated according to the homeostasis model assessment for IR (HOMA-IR) by the formula fasting insulin level (mUI/L) × fasting glucose level (mg/dL)/405. Subsequently patients were divided into two subgroups with respect to the HOMA-IR value—below and equal to or above 2.5.

### 2.2. Liver Histology

All CHC patients had liver biopsies performed with the Hepafix kit (B. Braun, Melsungen AG, Germany) as a part of the diagnostic routine before the antiviral therapy. Tissue samples were immediately divided into higher part for histopathological examination and the smaller one was stabilized in RNA*later* (Sigma-Aldrich, St. Louis, USA) and frozen at −80°C for further molecular procedures. Biopsy samples included at least eleven portal tracts and were examined by two pathologists. Histopathological features were assessed according to Scheuer's (necroinflammatory activity and fibrosis), Brunt's (steatosis), and Kleiner's (ballooning degeneration) scales [34–36].

### 2.3. Chemerin and Chemokine-Like Receptor 1 (CMKLR1) Expression in Liver Tissue

Total RNA was isolated from liver biopsy specimens of CHC patients using the RNeasy Mini Kit (Qiagen, Hilden, Germany). In addition to the standard procedure, RNase Free DNase Set (Qiagen, Hilden, Germany) was used to remove trace amounts of genomic DNA. RNA was quantified by measuring the absorbance at 260 and 280 nm (NanoDrop 1000 Spectrophotometer, Thermo Fisher Scientific, Wilmington, USA) and the integrity was assessed by electrophoresis in 1.2% agarose gel ethidium bromide stained. RNA isolates were used to cDNA synthesis with reverse transcription method using High Capacity RNA—to cDNA Kit (Applied Biosystems, Foster City, USA) according to manufacturers' instructions. Received cDNA was used to determine* chemerin* and* CMKLR1* genes expression level by real-time quantitative PCR (RT-Q-PCR) assay (TaqMan system).* Glyceraldehyde-3-phosphate dehydrogenase (GAPDH)* was used as housekeeping gene. TaqMan primers and probe for* chemerin*,* CMKLR1*, and* GAPDH* were bought as ready to use assays: Hs 01123775_m1 for chemerin, Hs 01386063_m1 for chemerin receptor (chemokine-like receptor 1, CMKLR1), and human GAPD endogenous control (FAM/MGB Probe, Nonprimer Limited) for GAPDH (Applied Biosystems, Foster City, USA). RT-Q-PCRs were performed in duplicates on the ABI PRISM 7300 Real Time PCR Detection System (Applied Biosystems, Foster City, USA), including negative control in all amplification reactions. Thermal cycling for both analyzed genes and* GAPDH* was initiated with an incubation step at 50°C for 2 min, followed by a first denaturation step at 95°C for 10 min, and continued with 40 cycles of 95°C for 15 s, 60°C for 1 min. The standard curves for a housekeeping gene* GAPDH* and the target genes were generated by serial dilutions of the control cDNA (equivalent to 1 *μ*g of total RNA) in four 2-fold dilution steps. The* chemerin* and* CMKLR1* expression levels were determined in every sample from the respective standard curve and divided by the* GAPDH* gene expression to obtain a normalized target value (relative expression level).

### 2.4. Statistical Analysis

The data were presented as mean ± SD. Differences between groups were examined through nonparametric tests (Mann-Whitney or Kruskal-Wallis) and linear correlation and logistic regression analysis using the Statistica software version 10.0. For all the analyses, statistical significance was determined for values of *P* < 0.05.

## 3. Results

Clinical and demographical data and the comparison of CHC patients with the control group have been summarized in Table 1. HOMA-IR but not serum glucose and insulin markedly increased in CHC patients compared to controls (Table 1).

Men and women entering the study group were similar according to age, diastolic blood pressure, and most biochemical parameters, but men had significantly higher BMI, waist circumference, systolic blood pressure, and GGT serum activity. General characteristics of the study participants are gathered in Table 1.

**Figure 1:**
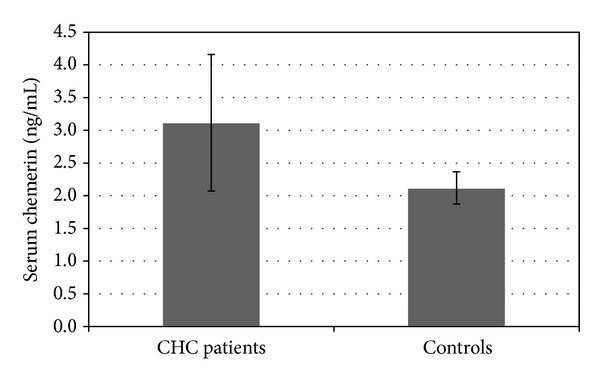
Serum chemerin in CHC patients and the control group.

Serum chemerin levels in CHC patients were significantly higher than in controls (3.12 ± 1.04 versus 2.11 ± 0.35 ng/mL; *P* < 0.001). There was no difference in serum chemerin between healthy men and women (2.16 ± 0.35 versus 2.07 ± 0.05 ng/mL; *P* = NS). The results were shown in Figure 1. There was no significant difference in serum chemerin between CHC male and female patients (2.85 ± 0.67 versus 3.37 ± 1.27 ng/mL, *P* = NS). Circulating chemerin level was not associated with any anthropometric or laboratory parameter except for AST activity (*r* = (−0.31), *P* = 0.04).

**Figure 2:**
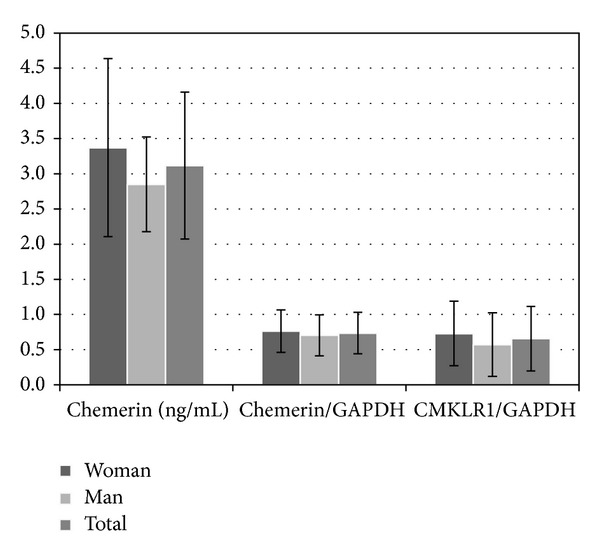
Serum chemerin concentration,* chemerin*, and* CMKLR1* liver tissue expression in CHC patients.

*Chemerin* hepatic expression reached 0.74 ± 0.30 in CHC patients and did not differ between men and women (0.70 ± 0.30 versus 0.76 ± 0.33, *P* = NS).* CMKLR1* relative expression in the whole study group reached 0.66 ± 0.46, with no significant difference between men and women (0.57 ± 0.45 versus 0.73 ± 0.46, *P* = NS). All the results were described in Figure 2.

Analysis of* chemerin* and* CMKLR1* tissue expression together with serum chemerin concentration in the whole study group and subsequently in male and female patients with respect to different BMI and HOMA-IR values is shown in Table 2.

The whole study group included 34 (54%) patients with BMI ≥25 kg/m^2^. BMI ≥25 kg/m^2^ was observed in 17 (50%) females and 17 (58.6%) males.* Chemerin* liver expression in women with BMI <25 kg/m^2^ was significantly higher than in those with higher BMI. There was no such difference in males (Table 2). Logistic regression analysis revealed significant relationship between BMI and hepatic* chemerin* expression in females (*P* < 0.05) but not in males (Table 3).

HOMA-IR ≥2.5 was found in 30 (47.6%) of CHC patients. Similar to BMI, higher insulin resistance was more frequent in men compared to women (51.7% versus 44.2%). Neither* chemerin* and its receptor gene liver tissue expression nor serum chemerin showed any influence on HOMA-IR abnormalities (Table 4).

Liver biopsy in CHC patients revealed necroinflammatory activity grade 1 in 11 (17.5%), grade 2 in 37 (58.7%), and grades 3-4 in 15 (23.8%) patients. Grade 2 was found in 58.6% of men and 58.8% of women, whereas grades 3-4 in 20.7% and 26.0%, respectively.

We did not find any significant relationship between necroinflammatory activity grade and* chemerin*,* CMKLR1* tissue expression, or chemerin serum levels (Table 5). Nevertheless, serum chemerin tended to be decreased in patients with more advanced inflammatory grade.

Liver fibrosis was found in all analyzed CHC patients. Portal fibrosis (stage 1) was observed in 27 (42.9%) and periportal fibrosis (stage 2) in 27 (42.9%), whereas bridging fibrosis/cirrhosis in 9 (14.2%) patients. There was no difference in fibrosis stage between men and women. None of these patients showed clinical features of liver cirrhosis at the time of study, but in 3 men and 3 women histopathological examination of liver tissue revealed stage 4 of fibrosis. Liver fibrosis of stage F1/F2/F3-4 was found in 12 (41.4%)/11 (37.9%)/6 (20.7%) men, and 15 (44.1%)/16(47.1%)/3 (8.8%) women, respectively. The highest serum chemerin was revealed in patients with stage 1, and it lowered along with fibrosis progression. However, the difference was significant only in the study group as a whole, but not in males or females separately (Table 6). On the other hand,* CMKLR1* liver tissue expression differed significantly only in females with different fibrosis stage (Table 6).

Liver steatosis was found in 30 (47.6%) of CHC patients, with grade 1 in 26 (41.3%) and grade 2 in 4 (6.3%) subjects. Due to a small number of patients having more extensive steatosis, further analysis was conducted to include patients with and without steatosis. Sex dependent analysis showed hepatic steatosis in 55.2% of males and 41.2% of females.* Chemerin* and its receptor gene tissue expression levels and serum chemerin concentrations did not differ between steatotic and nonsteatotic patients. The results are shown in Table 7.

Logistic regression analysis showed increased risk for steatosis along with lower* chemerin* liver tissue expression in all CHC patients, but there were no differences between men and women (Table 8).

Ballooning degeneration of hepatocytes was observed in 65.1% of CHC patients. Grade 2 was diagnosed more frequently in women compared to men (76.5% versus 51.7% of patients with ballooning degeneration). Levels of* chemerin* and its receptor gene hepatic expression and serum chemerin concentrations according to ballooning degeneration grading were collected in Table 9.

Neither serum chemerin nor tissue expression of its gene and* CMKLR1* in the liver was significantly associated with ballooning degeneration in the group of CHC patients (Table 10).

**Figure 3:**
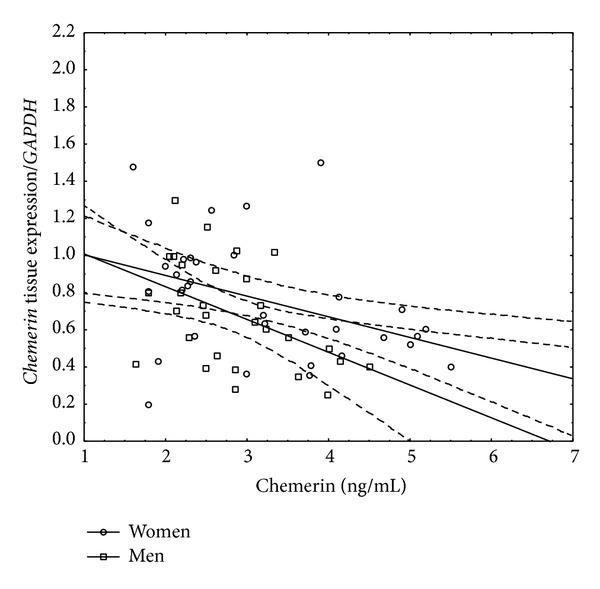
Linear correlation between serum chemerin concentrations and its gene expression in liver tissue in men and women with CHC.

**Figure 4:**
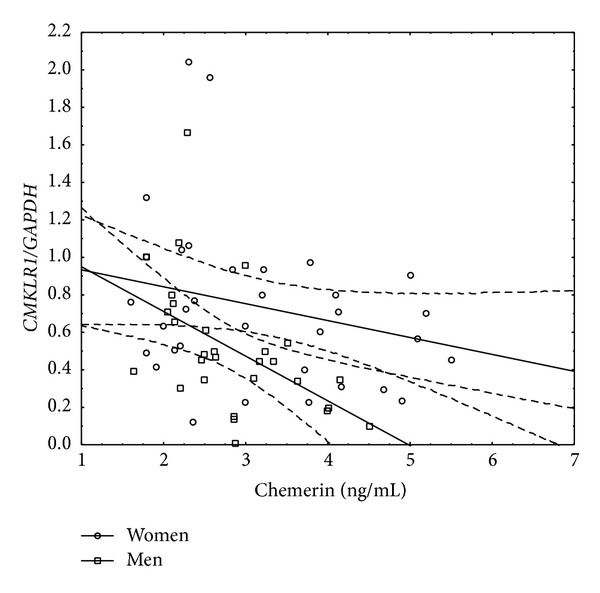
Linear correlation between serum chemerin concentrations and* CMKLR1* gene expression in liver tissue in men and women with CHC.

Finally we assessed the relationship between serum chemerin concentration and liver tissue* chemerin* or* CMKLR1* expression (Table 11). In the whole group of CHC patients and women, chemerin serum was inversely associated with its gene expression in the liver. However, this relationship was not more significant when assessed in men (Table 11, Figure 3). On the other hand, in men, serum chemerin was negatively associated with* CMKLR1* hepatic expression (Table 11, Figure 4).

## 4. **Discussion**

Previous reports indicate that liver injury may be associated with circulating chemerin [37–39] and the liver was supposed to contribute to serum levels [40]. In accordance with our previous study serum chemerin is strongly increased in patients with CHC compared to controls [33]. This is the first study which revealed substantial expression of chemerin and CMKLR1 in the liver of CHC patients. Previous studies assessed hepatic expression of chemerin and its gene in nonalcoholic fatty liver disease (NAFLD)/nonalcoholic steatohepatitis (NASH) [41] and animal models [9, 42]. Despite many metabolic aspects and some similarities in pathogenesis, it is difficult to compare CHC with NAFLD. Nevertheless, the role of some adipokines in pathogenesis and acceleration of CHC progression has been well established [31]. However, the direct impact of the virus on metabolic and inflammatory pathways must be considered. Obesity with accompanying metabolic syndrome with insulin resistance may directly influence levels of adipokines. A high amount of visceral adipose tissue may become an abundant source of adipokines, and therefore results of some studies may be unequivocal. Moreover, insulin resistance in obese individuals is primary metabolic, not viral, whereas in our study we would prefer to concentrate on virally derived metabolic abnormalities. Additionally, in obese CHC patients coexistence with NAFLD is common and exacerbates progression of viral disease by increasing insulin resistance and steatosis. Therefore, to avoid the possible influence of metabolic abnormalities associated with obesity and metabolic syndrome on adipokines profile, we decided to include only normal weight and overweight but not obese CHC patients. An additional aspect of the study is the choice of a homogeneous group of CHC patients consisting only of those infected with genotype 1b, in whom insulin resistance is the most distinct [22, 31]. HCV genotype 1b evokes insulin resistance by direct influence on intracellular insulin signaling pathway or by indirect mechanisms related with metabolic disturbances [22, 25]. Steatosis in genotype 1b infected patients results mainly from metabolic abnormalities. On the other hand, in genotype 3 infection steatosis is mainly viral derived, with less emphatic metabolic disturbances [25]. Therefore, it is difficult to compare the influence of both 1b and 3 genotypes on adipokines profile.

Necroinflammatory activity of different grade is a hallmark of chronic hepatitis including CHC. Due to the propagated pro- and/or anti-inflammatory action of chemerin in terms of tissue injury [15], we decided to evaluate the eventual relationship between hepatic* chemerin* and* CMKLR1* mRNA expression and necroinflammatory activity grade. Inflammation contributes to higher adipocyte chemerin synthesis but seems not to upregulate hepatocyte chemerin production. Proinflammatory agents such as interleukin- (IL-) 6 and tumor necrosis factor (TNF)*α*, which are upregulated in CHC [31, 43], had no effect on chemerin mRNA and cellular and soluble protein in primary human hepatocytes (PHH) [41, 44]. In PHH TNF*α* even lowered chemerin in cell supernatants without changing cellular levels [44]. A recent report indicated decreased serum IL-6 levels and reduced hepatic inflammatory cell invasion in CMKLR1−/− mice [45]. Thus, the chemerin-CMKLR1 system seems to be involved in tissue inflammation. In NAFLD* chemerin* liver expression was significantly associated with NAFLD activity score (NAS). Patients with defined NASH revealed markedly elevated hepatic* chemerin* expression compared to those with undefined or no NASH patients [41]. Our study did not find any relationship between hepatic* chemerin* or* CMKLR1* mRNA and inflammatory activity grade. In accordance with our previous reports serum chemerin level tended to be lower in patients with more advanced inflammatory activity grade [33, 38].

Higher levels of chemerin in hepatic venous serum compared to portal venous serum of patients with liver cirrhosis indicate that chemerin is released by the cirrhotic liver [11]. However, the question is whether this is the result of higher hepatic releasing or inappropriate clearance of circulating protein. In our study the highest concentration of serum chemerin was seen in patients with F1 stage, and it lowered along with fibrosis progression (*P* = 0.02), but we failed to detect significant difference with respect to* chemerin* hepatic expression in relation to various fibrosis stage.* CMKLR1* expression was significantly lower only in women with advanced fibrosis. Insulin resistance (IR) is one of the contributors to liver fibrosis in CHC. Chemerin was reported to enhance insulin-stimulated glucose uptake and insulin receptor substrate-1 tyrosine phosphorylation, suggesting that chemerin increases insulin sensitivity [46]. On the other hand chemerin was observed to induce synthesis of a potent fibrogenic agent—transforming growth factor- (TGF-) *β* in macrophages [47]. The limitation of the study is a low number of patients with bridging fibrosis or cirrhosis. Hence, the association of chemerin with fibrogenesis may not be excluded. Therefore, further studies with a higher number of patients with advanced fibrosis are necessary to establish exact expression of* chemerin* and* CMKLR1* in these cases. It should also shed some light on the role of serum chemerin as well as its gene and receptor expression in fibrosis progression.

Lipids are essential in the HCV life cycle; therefore, they must be accumulated in a sufficient amount in infected hepatocytes. There are well-evidenced experimental studies that show HCV core protein to be sufficient in evoking hepatic steatosis by triglycerides accumulation [28, 31]. In our study hepatic steatosis was observed in about half of analyzed CHC patients, which is in accordance with general observations [27, 28, 31]. There was no difference in serum chemerin, hepatic* chemerin*, or* CMKLR1* mRNA expression in CHC patients. However, logistic regression analysis pointed to hepatic* chemerin* as an important contributor of steatosis, seemingly playing a rather protective role.

In humans with NAFLD hepatic chemerin mRNA expression is positively associated with BMI and steatosis grade [41] and mRNA levels tend to be higher in patients with liver steatosis compared to controls [41, 44]. Interestingly, hepatic CMKLR1 protein is reduced in the liver of human subjects suffering from hepatic steatosis and becomes upregulated by adiponectin [16], suggesting a protective role of the receptor under conditions of liver steatosis. Similarly, in our study, lower hepatic expression of* chemerin* was a risk factor for more extended steatosis. The obtained result does not necessarily apply to HCV genotype 3 infected patients, in whom steatosis is mainly viral derived, whereas in genotype 1b infection steatosis results mainly from metabolic abnormalities [25, 31].

Hepatocytes ballooning degeneration is postulated to be related with fat droplets accumulation and concomitant cytoskeletal injury [48]. In our CHC patients this phenomenon was not associated with circulating chemerin concentration or with its gene and* CMKLR1* liver expression.

Insulin upregulates adipocyte chemerin whereas mRNA expression is not enhanced in PHH [49–51]. HCV evokes IR in the early stage of the infection and therefore increases the risk of the onset of T2DM in predisposed individuals. Some studies indicated that IR is associated with viral load and observed more likely in genotype 1 or 4 infection [31]. All these results point to a direct viral influence on IR independent of BMI and visceral adiposity and HCV itself may promote and exacerbate IR. The relationship between IR and HCV infection is complex and bidirectional. HCV induces steatosis and the latter could also induce and exacerbate IR. Similar to our previous studies [33], there was no association between serum chemerin and HOMA-IR. Also, hepatic* chemerin* and* CMKLR1* expression was not related to IR. The complex interplay between virus, steatosis, and insulin sensitivity may influence obtained results. However, further studies are necessary to elucidate chemerin influence on insulin sensitivity and hepatic steatosis in CHC.

Several studies found that serum chemerin is similar in males and females while others show that adipose tissue expression and serum levels are associated with gender suggesting that sex may also be relevant when studying expression of chemerin in NAFLD [11, 14, 52, 53]. Due to equivocal results we decided to compare hepatic expression of* chemerin* and* CMKLR1* in men and women with CHC. The present study confirmed our previous results, which did not show any difference of serum chemerin between males and females with CHC [33]. Also levels of* chemerin* and* CMLKR1* hepatic expression were similar in males and females.

Another novel and very intriguing finding of the present study was a negative association between serum chemerin and* chemerin* hepatic expression, which was significant in the whole group and in women, but not in men. These results were opposite to those obtained by Döcke et al. in NAFLD, who found serum chemerin to be positively associated with hepatic mRNA expression, when circulating chemerin was adjusted for body fat [41]. These observations point to white adipose tissue as a main source of chemerin in NAFLD patients. In our study which included normal-weight and overweight patients, the eventual amount of white adipose tissue might be significantly lower and could not serve as a plentiful source of chemerin.

*CMKLR1* hepatic expression was negatively associated with serum chemerin but only in men. Similar investigations in another group of patients with CHC have not been reported so far, so we could only speculate about the possible explanations.* Chemerin* and* CMKLR1* expressions were estimated in liver tissue homogenates; therefore, the cell type being the main source of these molecules is impossible to define. As mentioned above, circulating chemerin acts through its receptor, but it is still unknown if higher serum levels lead to lower receptor gene expression in liver and why only in men such a relationship could be important. On the other hand, if the circulating molecule achieves concentrations high enough for regulation of related processes, this in turn can lead to its gene suppression in target tissue. If and why this phenomenon is associated with CHC remain to be elucidated.

## 5. **Conclusions**

Our study, which focused on* chemerin* and* CMKLR1* expression, confirmed for the first time a marked expression of* chemerin* and its receptor,* CMKLR1*, in the liver of CHC patients and pointed to the possibility of chemerin pathway regulatory role in some pathogenetic aspects. Despite its documented role in inflammation,* chemerin* and its receptor gene expression showed no important influence on liver necroinflammatory staging. Lower* chemerin* liver tissue expression was a risk factor of steatosis development. The study was carried out using the homogenates of human liver tissue. Therefore, on the basis of the obtained results, it is not possible to define whether hepatocytes or other cell types, which are abundantly present in the liver, constitute the main source of* chemerin* and* CMKLR1* mRNA. Chemerin is activated by proteolytic processing, and assays to measure its local bioactivity have to be performed. Moreover, findings of sex-dependent* chemerin* and* CMKLR1* liver tissue expression point to possible impact of sex hormones or different adipose tissue localization on chemerin synthesis and its action. Pointing to a diverse impact of particular HCV genotypes on metabolic disturbances, it seems to be justifiable to compare* chemerin* liver expression in patients infected with different genotypes. Additional studies evaluating hepatic chemerin expression in other liver diseases are needed. Subsequent comparison with CHC patients would facilitate a better understanding of the exact role of this adipokine in pathogenesis of some liver diseases. Further research is necessary to clarify hepatic expression of* chemerin* and* CMKLR1* in CHC and function of finally synthesized proteins.

## Figures and Tables

**Table 1:** General characteristics of CHC patients and controls.

	Men *N* = 29 (46%)	Women *N* = 34 (54%)	*P* ^∗^	CHC patients *N* = 63	Control group *N* = 30	*P* ^∗∗^
Age (years)	48.2 ± 16.6	45.3 ± 12.8	NS	46.6 ± 14.6	47.9 ± 14.8	NS
Body mass index (kg/m^2^)	25.7 ± 2.6	24.0 ± 3.1	0.02	24.8 ± 3.0	23.9 ± 3.3	NS
Waist circumference (cm)	87.8 ± 11.2	79.4 ± 12.5	0.03	87.8 ± 11.2	85.5 ± 8.7	NS
Systolic blood pressure (mmHg)	135.0 ± 18.8	125.0 ± 12.5	0.02	129.0 ± 14.4	115.0 ± 10.5	NS
Diastolic blood pressure (mmHg)	79.1 ± 8.9	80.0 ± 9.5	NS	79.6 ± 9.0	77.1 ± 6.6	NS
Insulin (*μ*U/L)	10.6 ± 6.3	12.4 ± 11.8	NS	11.6 ± 9.6	10.0 ± 4.5	NS
Glucose (mg/dL)	95.1 ± 16.4	92.6 ± 15.8	NS	93.7 ± 16.0	80.8 ± 9.0	NS
HOMA-IR	2.5 ± 1.3	3.4 ± 3.5	NS	2.9 ± 2.7	2.0 ± 0.5	**0.03**
Bilirubin (*μ*mol/L)	15.1 ± 4.1	13.6 ± 11.2	NS	14.3 ± 8.7	9.7 ± 3.5	**0.02**
GGT (U/L)	104.8 ± 119.2	60.2 ± 34.5	**0.04**	80.7 ± 86.9	26.0 ± 5.5	**0.04**
ALT (U/L)	83.2 ± 50.8	98.8 ± 37.7	NS	75.4 ± 44.5	25.6 ± 4.3	**0.001**
AST (U/L)	54.2 ± 28.5	59.5 ± 22.5	NS	51.7 ± 25.3	24.1 ± 3.8	**0.004**
Total cholesterol (mg/dL)	200.9 ± 44.3	178.2 ± 54.6	NS	188.3 ± 50.9	174.6 ± 33.2	NS
LDL cholesterol (mg/dL)	84.2 ± 38.9	108.6 ± 51.5	NS	97.2 ± 47.2	94.5 ± 20.2	NS
HDL cholesterol (mg/dL)	51.2 ± 28.2	53.4 ± 21.3	NS	52.4 ± 24.2	44.0 ± 12.8	NS
Triglycerides (mg/dL)	134.8 ± 57.4	128.3 ± 63.00	NS	131.0 ± 60.1	139.0 ± 45.0	NS
CRP (mg/dL)	1.8 ± 1.1	1.6 ± 1.4	NS	1.7 ± 1.2	1.5 ± 1.4	NS
HCV viral load (IU/L)	2781433 ± 487426	1432246 ± 1976597	NS	2053301 ± 364614		

^∗^ *P*: men versus women; ^∗∗^ *P*: CHC patients versus controls.

**Table 2:** Serum chemerin concentration, *chemerin*, and *CMKLR1* tissue expression in CHC patients according to sex, BMI, and HOMA-IR.

	Men		Women		CHC patients	
	BMI < 25	BMI ≥ 25	BMI < 25	BMI ≥ 25	BMI < 25	BMI ≥ 25
Chemerin (ng/mL)	2.84 ± 0.67	2.85 ± 0.71	3.04 ± 0.83	3.56 ± 1.45	2.93 ± 0.73	3.23 ± 1.20
*Chemerin* tissue expression	0.70 ± 0.30	0.71 ± 0.29	**0.91 ± 0.31** ^∗^	**0.65 ± 0.25** ^∗^	0.81 ± 0.32	0.67 ± 0.27
*CMKLR1* tissue expression	0.65 ± 0.57	0.50 ± 0.31	0.79 ± 0.47	0.68 ± 0.46	0.72 ± 0.52	0.60 ± 0.40

	HOMA-IR < 2.5	HOMA-IR ≥ 2.5	HOMA-IR < 2.5	HOMA-IR ≥ 2.5	HOMA-IR < 2.5	HOMA-IR ≥ 2.5

Chemerin (ng/mL)	2.77 ± 0.83	2.83 ± 0.56	3.32 ± 1.52	3.35 ± 1.14	3.07 ± 1.26	3.08 ± 0.90
*Chemerin* tissue expression	0.58 ± 0.24	0.76 ± 0.37	0.84 ± 0.30	0.58 ± 0.22	0.73 ± 0.30	0.68 ± 0.31
*CMKLR1* tissue expression	0.48 ± 0.14	0.42 ± 0.33	0.84 ± 0.42	0.68 ± 0.58	0.68 ± 0.37	0.54 ± 0.47

^*∗*^BMI < 25 versus BMI ≥ 25 kg/m^2^; *P* < 0.05.

**Table 3:** Serum chemerin concentration, *chemerin*, and *CMKLR1* tissue expression-logistic regressions adjusted for BMI.

	Men			Women			CHC patients		
	Odds ratio	95% CI	*P*	Odds ratio	95% CI	*P*	Odds ratio	95% CI	*P*
Chemerin (ng/mL)	0.98	0.27–3.55	NS	0.72	0.35–1.46	NS	0.74	0.39–1.39	NS
*Chemerin* tissue expression	0.95	0.05–17.45	NS	30.65	1.14–823.14	**0.03**	4.96	0.69–7.11	NS
*CMKLR1 *tissue expression	2.28	0.32–16.12	NS	1.70	0.30–9.66	NS	1.85	0.53–6.43	NS

**Table 4:** Serum chemerin concentration, *chemerin*, and *CMKLR1* tissue expression-logistic regression adjusted for HOMA-IR.

	Men			Women			CHC patients		
	Odds ratio	95% CI	*P*	Odds ratio	95% CI	*P*	Odds ratio	95% CI	*P*
Chemerin (ng/mL)	0.86	0.20–3.75	NS	0.98	0.47–2.03	NS	1.99	0.54–1.82	NS
*Chemerin* tissue expression	0.14	0.01–4.32	NS	43.20	0.61–30.20	NS	1.74	0.20–15.50	NS
*CMKLR1 *tissue expression	2.85	0.05–165.99	NS	2.14	0.24–18.92	NS	2.34	0.45–13.24	NS

**Table 5:** Serum chemerin concentration, *chemerin*, and *CMKLR1* tissue expression in CHC patients with different necroinflammatory grade.

	Men				Women				CHC patients			
	1	2	3-4	*P*	1	2	3-4	*P*	1	2	3-4	*P*
Chemerin (ng/mL)	3.15 ± 0.76	2.85 ± 0.67	2.66 ± 0.69	NS	3.70 ± 0.85	3.87 ± 1.50	2.57 ± 0.57	NS	3.43 ± 0.80	3.32 ± 1.22	2.60 ± 0.59	NS
*Chemerin* tissue expression	0.76 ± 0.28	0.72 ± 0.34	0.60 ± 0.21	NS	0.84 ± 0.27	0.72 ± 0.32	0.79 ± 0.32	NS	0.80 ± 0.26	0.72 ± 0.32	0.72 ± 0.29	NS
*CMKLR1 *tissue expression	0.48 ± 0.24	0.62 ± 0.59	0.55 ± 0.30	NS	0.55 ± 0.22	0.67 ± 0.36	0.93 ± 0.65	NS	0.51 ± 0.22	0.65 ± 0.46	0.78 ± 0.56	NS

**Table 6:** Serum chemerin concentration, *chemerin*, and *CMKLR1* tissue expression in CHC patients with different fibrosis stage.

	Men				Women				CHC patients			
	1	2	3-4	*P*	1	2	3-4	*P*	1	2	3-4	*P*
Chemerin (ng/mL)	3.10 ± 0.56	2.69 ± 0.71	2.81 ± 0.77	NS	4.00 ± 1.43	3.02 ± 0.89	2.03 ± 0.17	NS	3.62 ± 1.21	2.86 ± 0.81	2.59 ± 0.74	**0.02**
*Chemerin* tissue expression	0.66 ± 0.27	0.71 ± 0.25	0.80 ± 0.43	NS	0.78 ± 0.33	0.73 ± 0.28	0.83 ± 0.38	NS	0.72 ± 0.30	0.72 ± 0.26	0.81 ± 0.38	NS
*CMKLR1 *tissue expression	0.55 ± 0.53	0.51 ± 0.29	0.73 ± 0.56	NS	0.69 ± 0.28	0.83 ± 0.62	0.47 ± 0.05	**0.04**	0.62 ± 0.41	0.70 ± 0.53	0.63 ± 0.45	NS

**Table 7:** Serum chemerin concentration, *chemerin*, and *CMKLR1* tissue expression with respect to the presence of hepatic steatosis.

	Men		Women		CHC patients	
	Present	Absent	Present	Absent	Present	Absent
Chemerin (ng/mL)	2.84 ± 0.70	2.86 ± 0.68	3.68 ± 1.55	3.09 ± 0.92	3.23 ± 1.22	2.99 ± 0.81
*Chemerin* tissue expression	0.62 ± 0.29	0.80 ± 0.27	0.69 ± 0.27	0.83 ± 0.32	0.65 ± 0.28	0.81 ± 0.29
*CMKLR1 *tissue expression	0.55 ± 0.50	0.59 ± 0.41	0.62 ± 0.29	0.82 ± 0.55	0.58 ± 0.40	0.72 ± 0.50

**Table 8:** Serum chemerin concentration, *chemerin*, and *CMKLR1* tissue expression-logistic regressions adjusted for steatosis.

	Men			Women			CHC patients		
	Odds ratio	95% CI	*P*	Odds ratio	95% CI	*P*	Odds ratio	95% CI	*P*
Chemerin (ng/mL)	1.06	0.29–3.88	NS	0.67	0.32–1.39	NS	0.80	0.44–1.43	NS
*Chemerin* tissue expression	10.72	0.43–267.07	NS	5.29	0.32–86.88	NS	7.45	0.96–57.99	**0.04**
*CMKLR1 *tissue expression	1.23	0.19–7.97	NS	3.16	0.42–23.82	NS	2.03	0.56–7.41	NS

**Table 9:** Serum chemerin concentration, *chemerin,* and *CMKLR1* tissue expression and ballooning degeneration grade.

Ballooning degeneration grade	Men		Women		CHC patients	
	0-1	2	0-1	2	0-1	2
Chemerin (ng/mL)	2.93 ± 0.95	2.82 ± 0.59	2.95 ± 0.68	3.91 ± 1.53	2.95 ± 0.75	3.26 ± 1.18
*Chemerin* tissue expression	0.50 ± 0.29	0.75 ± 0.28	0.81 ± 0.32	0.71 ± 0.27	0.73 ± 0.34	0.73 ± 0.27
*CMKLR1 *tissue expression	0.38 ± 0.21	0.64 ± 0.50	0.76 ± 0.48	0.74 ± 0.48	0.66 ± 0.45	0.68 ± 0.48

**Table 10:** Serum chemerin concentration, *chemerin*, and *CMKLR1* tissue expression-logistic regression adjusted for ballooning degeneration of hepatocytes.

	Men			Women			CHC patients		
	Odds ratio	95% CI	*P*	Odds ratio	95% CI	*P*	Odds ratio	95% CI	*P*
Chemerin (ng/mL)	1.28	0.29–5.57	NS	0.45	0.18–1.15	NS	0.72	0.38–1.39	NS
*Chemerin* tissue expression	0.02	0.0001–3.11	NS	3.48	0.20–60.23	NS	0.93	0.12–7.08	NS
*CMKLR1 *tissue expression	0.11	0.002–8.09	NS	1.13	0.20–6.33	NS	0.90	0.25–3.27	NS

**Table 11:** Linear correlation between serum chemerin and *chemerin* or *CMKLR1 *tissue expression.

	chemerin (ng/mL)		
	Men	Women	CHC patients
*Chemerin* tissue expression	*r* = −0.37	*r* = −0.54	*r* = −0.41
	*P* = NS	***P *** = 0.006	***P *** = 0.004
*CMKLR1 *tissue expression	*r* = −0.44	*r* = −0.26	*r* = −0.21
	***P *** = 0.04	*P* = NS	*P* = NS
